# The Ibogaine Experience Scale (IES): Development and psychometric properties of a multidimensional measure of ibogaine’s subjective effects

**DOI:** 10.1371/journal.pone.0333296

**Published:** 2025-10-13

**Authors:** Francisco González Espejito, Laura Esteban Rodríguez, Eduardo J. Pedrero Pérez, Jonathan Dickinson, Maja Kohek, Rafael Guimaraes dos Santos, Jaime Hallak, Miguel Ángel Alcázar-Córcoles, Breanna Lee Morgan, José Carlos Bouso

**Affiliations:** 1 Department of Biological and Health Psychology, Faculty of Psychology, Autonomous University of Madrid, Madrid, Spain; 2 Psychiatry Service, 12 de Octubre University Hospital; Health Research Institute Hospital 12 de Octubre (imas12), Madrid, Spain; 3 Faculty of Psychology, Complutense University of Madrid, Madrid, Spain; 4 Department of Psychobiology, National Distance Education University (UNED), Madrid, Spain; 5 Ambio Life Sciences Inc, Vancouver, Canada; 6 International Center for Ethnobotanical Education, Research & Service (ICEERS), Barcelona, Spain; 7 Medical Anthropology Research Center (MARC), Department of Anthropology, Philosophy and Social Work, University Rovira i Virgili, Tarragona, Spain; 8 Department of Neurosciences and Behavior, University of São Paulo, São Paulo, Brazil; 9 National Institute of Translational Science and Technology in Medicine (INCT-TM), CNPq, Ribeirão Preto, Sāo Paulo, Brazil; Universitas Islam Negeri Raden Intan Lampung, HUNGARY

## Abstract

Ibogaine, an indole alkaloid derived from the root bark of *Tabernanthe iboga*, has long been used in traditional Bwiti healing rituals and shows promise for treating opioid dependence and neurological conditions, but existing psychometric tools fail to capture its distinctive subjective/oneiric (dream-like) effects. To address this gap, we developed the 70-item Ibogaine Experience Scale (IES) through an iterative process informed by a prior qualitative study (n = 20) that identified eight experiential domains. A preliminary 144-item version was completed on site with a mobile device within 48 hours of treatment by 499 participants across two clinical settings—cohort neuropsychiatric treatments (n = 381) and substance use disorder treatments (n = 118). We employed exploratory graph analysis, parallel analysis on polychoric correlations, and iterative item‐reduction (Gulliksen’s Pool, MIREAL, MSA) to refine the scale. Semi-confirmatory factor analysis used Robust Unweighted Least Squares (RULS) with LOSEFER correction, oblimin rotation, and multiple fit indices (CFI, NNFI, GFI, AGFI, RMSR, WRMR). Cronbach’s α, McDonald’s ω, H indices, EAP reliability, FDI, ORION, SR, and EPTD assessed internal consistency and factorial quality. The final structure comprises seven factors—Narrative and symbolic visions; Visual changes; Discomfort and challenge; Cosmic/Archetypal Visions; Introspection and personal transformation; Somatosensory hypersensitivity and physiological activation; Dissociation—explaining 53.9% of variance, with excellent fit (CFI = .991; GFI = .983; RMSR = .041; WRMR = .038) and high internal consistency (α = .948; ω = .946; subscale ω = .65–.91). Two subscales exhibited small gender effects. The IES provides a reliable, phenomenologically rich instrument for quantifying ibogaine’s distinctive subjective effects. It supports research and clinical assessment by capturing the multidimensional, oneiric/dream-like nature of the ibogaine experience. Future work should confirm this structure in independent, culturally diverse cohorts and explore predictive links between IES domains and therapeutic outcomes.

## Introduction

Ibogaine is a psychedelic indole alkaloid found in high concentrations in the root bark of *Tabernanthe iboga* (iboga), a perennial shrub endemic to Gabon and surrounding regions of the Central African rainforest. The plant itself has been used for centuries in the spiritual practice of the Bwiti, where it serves as a central sacrament in rituals of initiation and healing [1]. Within this traditional context, music, mantras, perfumes, colors, movement, and intricate symbolism are used to intentionally strengthen and guide the subjective dreamlike (oneiric) experience of *banzis* (initiates) toward a variety of beneficial ends.

Ibogaine has a complex pharmacological profile. Unlike classical psychedelics such as LSD or psilocybin, which exert their effects primarily via strong agonism at the 5-HT2A receptor, ibogaine and its metabolite noribogaine display very low affinity for this receptor and do not produce the typical serotonergic hallucinogenic effects—evidenced by the absence of the head-twitch response in animal models. Instead, ibogaine’s psychoactive and therapeutic effects are mediated through a multifaceted pharmacology, including kappa-opioid receptor agonism, NMDA receptor antagonism, serotonin and dopamine transporter inhibition, and enhancement of neurotrophic factors like brain-derived neurotrophic factor (BDNF) and glial cell-derived neurotrophic factor (GDNF). Notably, noribogaine promotes structural neural plasticity, increasing dendritic complexity in a manner comparable to ketamine. These properties support its classification as an atypical psychedelic, with anti-addictive effects emerging from synergistic modulation across diverse neural systems rather than through classical serotonergic pathways [2].

In the West, extracted and purified ibogaine has been used for decades within medical and holistic contexts to mitigate withdrawal and cravings from opioids [3–5]. More recently, it has been used to reverse symptoms of traumatic brain injury (TBI) in combat veterans [6,7]. While most research has focused on the physiological outcomes of ibogaine’s unique pharmacology [3,8], studies frequently reveal the importance that subjects place upon their subjective experience when describing their overall healing process [9–13].

Precisely, one of the challenges of investigating the influence that ibogaine’s subjective effects have on patient outcome has been the failure of existing psychometric tools to quantify its distinctive phenomenology. Past studies investigating ibogaine outcomes have employed measurements like the Mystical Experience Questions (MEQ), which was designed to assess the very different effects of psilocybin and other classic psychedelics. However, researchers have commented on the obvious misalignment between the items measured by these instruments and the ibogaine experience, suggesting that “it may be that ibogaine’s unique properties require the development of an instrument sensitive to its oneiric [dream-like] effects” [10, (p162)].

The Ibogaine Experience Scale (IES) was developed to address the need for a state-specific measure of the distinctive phenomenology of ibogaine. This paper outlines the rationale, development, and psychometric properties of the IES, designed to facilitate a more accurate understanding of the experiential dimensions associated with ibogaine treatment.

## Materials and methods

### Scale development

**Fig 1:**
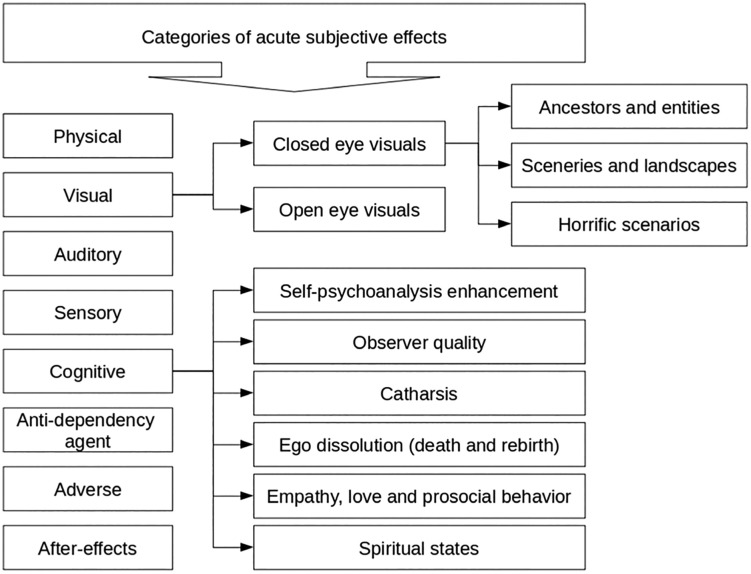
Theoretical categories of acute subjective effects of ibogaine consumption (retrieved from Kohek and colleagues [13, (p 98)].

The development of the IES was based on the prior qualitative study by Kohek and colleagues [13], which identified key dimensions of the acute subjective experience of ibogaine consumption (Fig 1). This study involved in-depth, semi-structured interviews with 20 individuals who had recently undergone ibogaine treatment, primarily for addiction recovery or personal growth. The interviews, conducted between February and April 2016, were analyzed using grounded theory methodology. This inductive approach enabled the researchers to derive a conceptual framework of eight primary categories of effects—physical, sensory, visual, cognitive, auditory, adverse, anti-dependency, and after-effects—and ten additional subcategories addressing specific experiential phenomena such as ego dissolution, empathy, and spiritual experiences [13].

These categories served as the conceptual basis for developing the IES and represent a broad spectrum of effects reported by participants. The physical effects category includes reports of changes in bodily perception, such as weight, temperature, coordination, and gastrointestinal sensations like nausea or constipation. Some participants also described less common experiences, such as feelings of electricity running through the body, tremors, and altered heart rhythms. The sensory effects category covers changes in perception of light, sound, smell, taste, and touch, as well as how sensitive participants felt to these stimuli. It also includes heightened awareness of senses and occurrences of synesthesia (e.g., feeling sound or seeing music). The visual effects category refers to open- and closed-eye imagery, such as geometric patterns, fractals, and visual alterations in the environment, including changes in color or the appearance of objects, people, or even entire scenes from an internal reality, rather than the external world. The cognitive effects category reflects altered thought processes, such as introspection, self-reflection, and enhanced capacity to focus, including the feeling of ego dissolution and insights into personal issues or past events.

In addition to these, participants reported challenging or adverse effects, such as fear, confusion, existential distress, and anxiety related to death or the loss of self. These were grouped into a distinct category to capture the more difficult aspects of the experience. The category of anti-dependency effects and after-effects was formed from reports highlighting ibogaine’s therapeutic potential, including reduced cravings, diminished withdrawal symptoms, and sustained psychological benefits such as emotional clarity, decreased anxiety, and enhanced optimism. Participants also reported on the personal meaning and perceived value of their experience, which informed items assessing satisfaction, integration, and the likelihood of recommending the treatment to others.

These categories informed the construction of scale items that aim to quantify the diverse and multidimensional nature of the ibogaine experience, while remaining grounded in the subjective reports of those who have lived it. To ensure that the full range of these effects—both challenging and therapeutic—were adequately captured, the items included in the questionnaire were developed by the authors drawing also on extensive familiarity with the subjective effects of iboga and ibogaine; years of informal interviews with patients describing their experiences in detail; and case studies exploring specific experiential phenomena such as perceived communication with the medicine or with entities encountered during the session [14].

Guided by this conceptual structure, a preliminary version of the IES was developed to translate these qualitative categories into quantifiable self-report items. The item pool was generated by a group of authors of the present manuscript—JD, MK, RGdS, JH, MÁAC, and JCB—who collaboratively wrote and selected items to represent the full range of the ibogaine experience. Each major category was represented by a minimum of five items, resulting in an initial set of 130 items. In addition, 11 general items were included to assess participants’ overall impression of the experience, and 3 supplementary questions captured temporal aspects, such as when the peak occurred, how long the intense effects lasted, and the total duration of the experience—bringing the total to 144 items.

All items were designed using a 4-point Likert scale format to assess the intensity of the experience (0 = None; 1 = Slightly; 2 = Moderately; 3 = Strongly). This structure was chosen to provide sufficient granularity without overwhelming respondents, allowing for nuanced measurement of diverse experiential domains while maintaining feasibility in terms of completion time. Additionally, 11 items were included to evaluate participants’ overall experience with ibogaine consumption in general terms. Three additional questions were designed to characterize the timing of the experience: (“The most intense effects occurred at (select the correct answer from when you took your first dose)”; “The most intense effects lasted (select the correct answer from when you took your first dose)”; “How many hours lasted the entire experience, from the first dose used?”) (see S1 File).

### Participants

Participants in this study were clients of Ambio Life Sciences, a treatment center located in Tijuana, Mexico. This center primarily serves individuals seeking to overcome substance use disorders, but also treats clients with psychological or neurological conditions such as mood disorders, traumatic brain injuries, and neurodegenerative diseases. Treatment was provided in a non-medical but supported setting, with staff and facilitators accompanying participants throughout the experience.

Ibogaine was administered as part of the center’s usual therapeutic practices, not as an intervention for this study; the two routine treatment settings are described here solely to provide context on the environments in which participants ingested the substance. In the first model, a cohort approach, groups of 4–6 individuals (typically without active substance use disorders) underwent treatment simultaneously; this setting accounted for most participants (n = 381; 76.4%). In the second model, used for opioid detoxification, individuals with polysubstance use (including opioids, amphetamines, cocaine, and alcohol) received treatment individually after medical stabilization (n = 118).

A total of 505 participants completed the preliminary version of the Ibogaine Experience Scale (IES). After excluding six cases due to incomplete responses, the final sample consisted of n = 499. Participants were recruited using a non-probabilistic convenience sampling method. The sample was diverse in terms of demographics, including age, country of origin, and educational background, ensuring the scale’s validity could be assessed across different groups. The mean age of the sample was 44 years (SD = 9.2), and the majority of participants were male (80.96%). Detailed demographic characteristics are presented in Table 1.

**Table 1:** Sample descriptives.

		Males	Females	DK/NA	Total
	**n**	404	94	1	499
**Age**	**Mean**	43.9	44.8		44
	**Standard deviation**	9.1	9.5		9.2
	**Rank**	18 - 71	23 - 69		18 - 71
**Highest level of education completed**	**Primary school**	1	1		2
	**High school**	121	20		141
	**University degree**	190	47		237
	**Postgraduate or doctorate degree**	92	26		118
**Country of birth**	**U.S.A.**	365	72	1	438
	**Canada**	15	8		23
	**Mexico**	4	4		8
	**European Union**	7	3		10
	**Other European Countries**	5	2		7
	**Asian Countries**	5	2		7
	**Central and South American Countries**	4	1		5
	**African Countries**	–	1		1
**Current country of residence**	**U.S.A.**	380	79	1	460
	**Canada**	12	7		19
	**European Union**	3	3		6
	**Other European Countries**	1	–		1
	**Central and South American Countries**	7	5		12
	**New Zealand**	1	–		1

### Procedure

The recruitment period for the study spanned from October 9, 2020, to May 17, 2024. Participants were invited to complete the questionnaire after their ibogaine session, and participation was entirely voluntary and anonymous. The questionnaire was distributed in two ways: (1) through informational posters with QR codes linking to the digital form, placed in the treatment facilities, and (2) via direct links shared in chat groups used to coordinate logistics for each treatment cohort.

To facilitate completion, dedicated time was carved out on the evening of the fourth day of the treatment program, providing clients with a quiet and supported setting to complete the questionnaire. Most participants filled it out during this session, while others completed it within one or two days after leaving the center. Clinic hosts encouraged completion within 48 hours post-treatment, and participants used their own mobile devices to access the online form.

Participants were informed that their responses would remain anonymous and that participation would not affect their treatment in any way. No incentives were offered. Written informed consent was obtained from all participants before completing the form. The study was reviewed and approved by the Ethics Committee of the Autonomous University of Madrid, Spain (reference: CEI-66–1174).

### Inclusivity in global research

Additional information regarding the ethical, cultural, and scientific considerations specific to inclusivity in global research is included in the Supporting Information (S7 File).

### Data analysis

**Fig 2:**
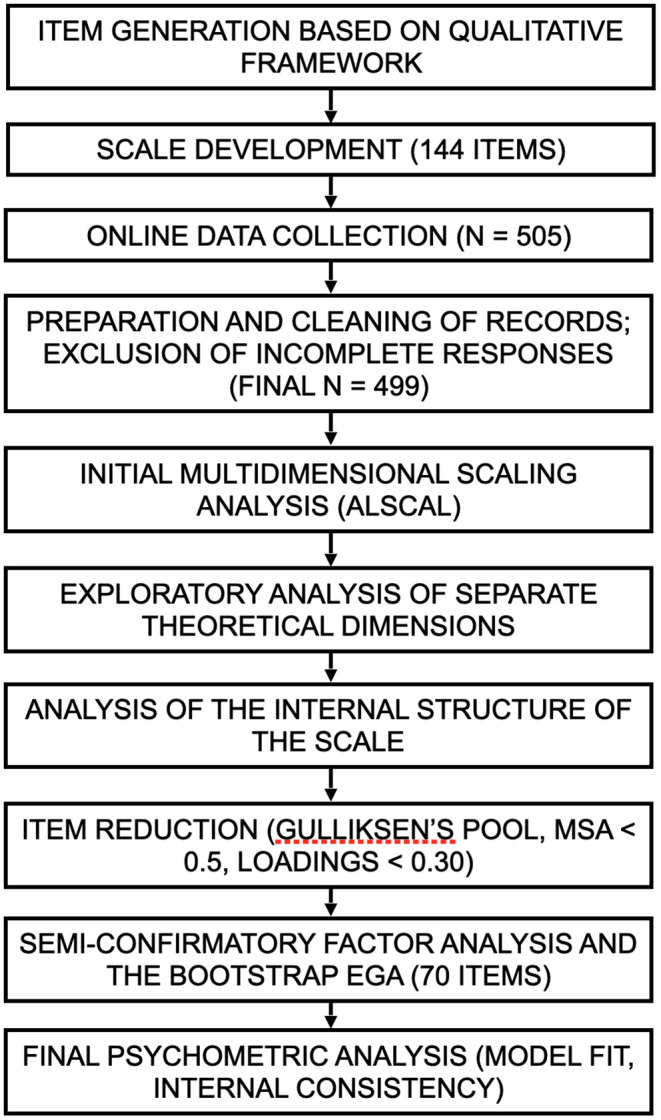
Schematic of the phases in the psychometric analysis.

Following data collection, the database was anonymized to remove all information that could potentially identify individual participants. Access to the complete, non-anonymized dataset was limited to two authors (JD and JCB) to safeguard confidentiality and comply with ethical standards. The anonymized dataset is provided in the Supporting Information (S8 File). Data were accessed for research purposes on July 3, 2024. The subsequent analysis steps, culminating in the evaluation of the instrument’s psychometric properties, are schematically depicted in Fig 2.

First, scores for each theoretical dimension were estimated, and initial multidimensional scaling analysis was conducted using ALSCAL. This allowed visualization of the conceptual proximity between items and assessment of their alignment with the proposed theoretical dimensions. The resulting configuration was reviewed by the research team, which confirmed its theoretical coherence. Since the “Antidependency-agent” dimension applied exclusively to participants who used ibogaine for the treatment of substance use disorder (*n* = 118), it was excluded from subsequent analyses and evaluated independently for potential inclusion in clinical applications specific to substance use disorder.

A preliminary item-level analysis was performed for each dimension to ensure adequate measurement properties, including appropriateness, fit, replicability, factorial quality, and internal consistency. To examine the dimensional structure of the IES, we applied several techniques: parallel analysis with resampling and principal components extraction based on the mean eigenvalue criterion and polychoric correlations [15], as well as parametric bootstrap exploratory graph analysis (EGA) with 500 iterations, using graphical LASSO regularization and the Louvain community detection algorithm [16]. Graphical outputs for both procedures—the parallel analysis and the bootstrap EGA—are provided in S4 File based on polychoric correlations (PC and FA methods) and S5 File, respectively, to visually illustrate the factor retention decisions. Given the non-normal distribution of the items, parallel analysis based on polychoric correlations is considered particularly appropriate [17]. In turn, EGA has demonstrated superior performance in detecting dimensionality when moderate inter-factor correlations are present [16]. The number of factors was determined based on the joint results of parallel analysis and bootstrap EGA.

Next, an exploratory factor analysis (EFA) was conducted separately for each theoretical dimension using FACTOR 12.06.07 [18]. Given that the data violated multivariate normality [19] and considering the Likert-scale nature of the items, a polychoric correlation matrix was employed, following methodological recommendations by Lloret-Segura and colleagues [20]. The number of dimensions to retain was determined using an optimized parallel analysis [21], and parameter estimation was performed via Robust Unweighted Least Squares (RULS) with the LOSEFER empirical correction applied to the robust chi-square [22].

For each individually analyzed dimension, multiple estimators were obtained to evaluate the internal structure quality of the dimension and its constituent items. Measure of Sampling Adequacy (MSA) values above 0.50 were deemed acceptable, as lower values suggest the item does not measure the same domain [23]. A Mean of Item Residual Absolute Loadings (MIREAL) value below 0.300 was interpreted as indicating the data could be treated as essentially unidimensional [24]. Additionally, the Goodness of Fit Index (GFI) and Generalized H Index were assessed, with values greater than 0.80 indicating a well-defined latent variable. The quality of factorial scores was examined using the Sensitivity Ratio (SR, recommended >2) and Expected Percentage of True Differences (EPTD, recommended > 90%). Internal consistency for each theoretical dimension was estimated through standardized Cronbach’s alpha (αs) and McDonald’s omega (ω). For transparency and to facilitate interpretation, a brief explanation of each psychometric statistic reported (e.g., MIREAL, FDI, ORION, EPTD, SR, H-index) is provided in the Supporting Information (see S6 File).

Following this preliminary analysis of the theoretical dimensions individually, the scale’s internal structure was analyzed globally, requiring item reduction. For this purpose, Gulliksen’s Pool with nonlinear parameterization was employed, following criteria from Lorenzo-Seva and Ferrando, and Ferrando and colleagues [23,25]. Suggested items were reviewed, Heywood cases were checked, and items excluded by the Pool based on Overall Item Threshold (OIT) and Overall Item Slope (OIS) were compared with those flagged as duplicates by the EREC (Expected Residual Correlation Direct Change Index) to reduce redundancy. Additionally, items with 95% confidence intervals for MSA below 0.50 were removed. After this initial item elimination round, a semi-confirmatory factor analysis was conducted using polychoric correlation matrices with RULS extraction, as previously justified. Oblimin rotation, an oblique method, was applied due to inter-factor correlations, enhancing interpretability, in line with recommendations by Schmitt and Sass [26], who advocate for oblique rotation when latent constructs are expected to be correlated. The final number of factors was determined via optimized parallel analysis, and items with factor loadings below 0.30 were excluded. This process yielded a final 70-item scale.

Subsequently, the semi-confirmatory factor analysis was repeated with this reduced 70-item version using the same methods described earlier. To assess the suitability of the factorization process, Kaiser-Meyer-Olkin (KMO) values above 0.80 and a statistically significant Bartlett’s test of sphericity (p < 0.05) were used as indicators, following Kaiser’s recommendations [27]. Since the correlation matrix was not positive definite, the Sweet Smoothing algorithm proposed by Lorenzo-Seva and Ferrando was applied [28].

The model fit was assessed using multiple indices: Non-Normed Fit Index (NNFI), Comparative Fit Index (CFI), Goodness of Fit Index (GFI), and Adjusted Goodness of Fit Index (AGFI). The following cutoff criteria were applied: CFI and NNFI >0.90 (good fit) and >0.95 (excellent fit) [29–34]; GFI ≥ 0.89 [35]; and AGFI >0.80 [36]. Additionally, residual distribution was examined using the Root Mean Square Residual (RMSR), with an expected mean value of 0.0448 for acceptable models according to Kelley’s criterion [37], and the Weighted Root Mean Square Residual (WRMR), for which values below 1.0 indicate good fit [38].

Factorial quality was determined through the H index (>0.80) for construct replicability, alongside other precision indicators such as the Factor Determinacy Index (FDI > 0.90), marginal reliability ORION (>0.80), Sensitivity Ratio (SR > 2), and Expected Percentage of True Differences (EPTD >90%) [24]. Score reliability was estimated using the Expected A Posteriori Scores (EAP) method. Communalities were considered acceptable if exceeding 0.15 and adequate if surpassing 0.50 [39,40]. Factor loadings above 0.40 were accepted, while values around 0.60 were deemed adequate [39].

The internal consistency of each scale was evaluated following Campo-Arias and Oviedo’s recommendations [41] using Cronbach’s alpha (α) [42] and McDonald’s omega (ω) [43]. For interpretation, a minimum threshold of ω ≥ 0.80 was established [44], and α values above 0.70 were considered acceptable [41].

The Antidependency-agent subscale was independently analyzed in the subsample that received ibogaine for the treatment of substance use disorder (n = 118). This 5-item subscale was examined using the methods described for individual scale analyses, including optimized parallel analysis to determine the number of factors to retain, along with estimators to assess unidimensionality and internal structure quality. Similarly, the global experience evaluation subscale -which measured overall satisfaction with the ibogaine experience rather than specific effects- was analyzed separately. The same analytical framework was applied, supplemented by a descriptive analysis of responses to the three items assessing individual experiences.

Once the factorial structure was established, scores were calculated for each scale. To explore potential differences in subjective experiences based on participants’ self-reported gender, a Multivariate Analysis of Covariance (MANCOVA) was conducted, controlling for age as a covariate. The magnitude of differences was quantified using omega-squared (ω²), an effect size estimator that adjusts for the positive bias inherent in eta-squared (η²), providing a conservative measure of variance explained by the independent factor.

## Results

### General questionnaire

The results of the EFA applied individually to each theoretical dimension generally showed adequate indicators. As shown in Table 2, GFI values exceeded 0.95 for most dimensions, except for the *Mood effects* dimension. Similarly, the H index for each dimension suggested well-defined latent variables (H ≥ .80) across all dimensions except the physical and sensory effects subscales. MIREAL values were below 0.3 in most cases, indicating that the data could be treated as essentially unidimensional, though higher values were observed for the sensory effects and *Mood effects* subscales. Regarding factorial score quality, Sensitivity Ratio (SR) and Expected Percentage of True Differences (EPTD) values were adequate for most dimensions, except the physical and sensory effects subscales, which also showed lower internal consistency values for standardized Cronbach’s alpha and McDonald’s omega.

**Table 2:** Properties of the obtained theoretical dimensions.

Dimension	No. of items	MSA (normed)	GFI	MIREAL	H-latent	H-observed	SR	EPTD	α_s_	ω
**Physical effects**	8	.73 −.83	.97	.28	.76	.68	1.8	87.6	.76	.76
**Sensory effects**	6	.70 −.87	.97	.32	.78	.70	1.9	88.2	.77	.78
**Visual effects open eyes**	6	.78 −.89	.99	.28	.86	.79	2.5	90.9	.84	.85
**Visual effects closed eyes**	6	.76 −.93	.99	.22	.91	.80	3.1	92.7	.86	.86
**Ancestors and entities**	5	.80 −.93	.99	.22	.95	.84	4.2	95.1	.91	.91
**Sceneries and landscapes**	6	.80 −.90	.99	.24	.89	.77	2.8	91.9	.85	.86
**Horrific scenarios**	4	.61 −.79	.99	.28	.86	.86	2.5	90.8	.67	.70
**Self-psychoanalysis enhancement**	8	.68 −.92	.98	.22	.90	.82	2.9	92.3	.83	.84
**Empathy, love and prosocial behavior**	5	.68 −.88	.99	.29	.91	.77	3.2	93.0	.86	.87
**Catharsis**	7	.90 −.96	.99	.15	.95	.79	4.4	95.4	.92	.92
**Observer quality**	5	.74 −.79	.99	.27	.84	.77	2.3	89.9	.83	.83
**Ego dissolution**	10	.63 −.85	.95	.27	.86	.76	2.5	90.8	.83	.83
**Spiritual states**	7	.72 −.91	.98	.29	.92	.83	3.5	93.7	.89	.89
**Auditory effects**	6	.64 −.89	.99	.25	.88	.75	2.7	91.6	.84	.84
**Mood effects**	6	.59 −.75	.86	.39	.99	.75	8.6	98.6	.63	.72

NOTE: MSA = Measure of Sampling Adequacy; GFI = Goodness of Fit Index; MIREAL = Mean of Item REsidual Absolute Loadings; H = Generalized H (G-H) Index; SR = Sensitivity Ratio; EPTD = Expected Percentage of True Differences; αs = Standardized Cronbach Alpha; ω = McDonald Omega.

In the analysis of the scale’s global internal structure, it was identified that the correlation matrix was not positive definite, leading to item review and reduction following the methodology’s procedures. After this process, a final 70-item version was obtained. Although the correlation matrix did not achieve positive definiteness, a single negative eigenvalue was observed, with a maximum value of −.017 and Sweet Smoothing algorithm affected only 0.4% of the covariance. To address this issue, the Sweet Smoothing algorithm proposed by Lorenzo-Seva and Ferrando [28] was applied, affecting only 0.4% of the covariance. The resulting polychoric correlation matrix showed a determinant below.001, an excellent Kaiser-Meyer-Olkin value (KMO = .922), and a significant Bartlett’s test of sphericity (χ² = 5455.2; df = 2415; *p* < .001). Measure of Sampling Adequacy (MSA) values for all items ranged between.750 and.962, exceeding the.50 threshold.

Regarding the assessment of dimensionality, parallel analysis indicated the presence of 11 latent factors and 7 principal components. In parallel, the bootstrap exploratory graph analysis (EGA) identified a 7-factor structure as the most frequently replicated solution across the 500 bootstrap samples (35.8%). Additional solutions with 8, 5, 6, and 9 dimensions were also observed, appearing in 23.6%, 18.6%, 17.8%, and 4.2% of the replicas, respectively, pointing to a moderate degree of dimensional stability. Considering that both parallel analyses based on polychoric correlations and EGA are recommended approaches when working with non-normal data and interrelated factors, the 7-factor solution was retained as the most theoretically and empirically supported. This structure accounted for 53.9% of the total explained variance. Communalities ranged between.19 and.73, while factor loadings varied from.27 to.84 in their respective factors (Table 3). The H index exceeded.80 for all factors, indicating well-defined latent variables and construct replicability [24]. Similarly, quality and efficacy indicators for factorial score estimates were adequate across all seven factors (FDI ≥ .90; ORION marginal reliability > .80; SR > 2; EPTD > 90%). The EAP (Fully Informative Prior Oblique EAP scores) estimates and ORION reliability indices reached values equal to or greater than.90 for all factors, reflecting high precision and measurement reliability.

**Table 3:** Communalities and factor loadings.

Item	NSV	VC	DC	C/AV	IPT	SHPA	DI	h²
17. Did you see dream-like sequences, with visions moving, changing or transforming one into the other?	.77	.14	.02	.00	.10	−.02	−.05	.73
18. Did any of these sequences involve characters or story-like scenes?	.69	.08	.06	.08	.12	.03	−.08	.67
16. Did you have visons with closed eyes (e.g., colors, lights, geometrical patterns, fractals)?	.57	.26	−.03	−.06	−.04	−.03	.16	.52
21. Did these dreams have a cartoonish or exaggerated quality?	.52	.13	.03	.03	−.05	.05	.10	.40
22. Did you see faces or masks?	.51	.20	−.06	.19	−.02	−.02	.08	.48
23. Did any of these others interact or communicate with you?	.47	−.02	.02	.26	.33	.09	−.10	.66
24. Did any of these interactions feel impactful or meaningful?	.45	−.01	.06	.25	.44	−.03	−.10	.73
19. Did you see scenes that repeated themselves?	.45	.16	.06	.07	−.06	.16	−.03	.37
25. Did you receive any specific information or insights from these interactions?	.44	−.09	.02	.25	.44	.01	−.05	.67
31. Did these images and visions have meaningful messages associated with them?	.38	−.06	−.02	.38	.26	.06	−.03	.58
20. Did you have paradoxical images or visions (e.g., light-dark, bad-good, life-death)?	.35	.16	.32	.26	.09	−.04	.04	.58
14. Did objects, people, or shadows take on the appearance of other things?	.17	.71	.04	−.06	.03	−.11	.07	.61
12. Did you perceive changes in the colors of the environment (e.g., more vibrant or vivid than usual, or black & white)?	.02	.70	−.01	−.03	.14	.14	−.08	.61
11. Did you see patterns or details in the external environment that you could not see before?	.00	.65	−.06	.06	.11	.13	−.08	.55
13. Did you see trails of light or other visual distortions (e.g., undulating walls, geometrical patterns, fractals)?	.07	.64	.05	−.05	.02	.00	.11	.50
15. Did you see visions of things or characters appear in the room with your eyes open?	.14	.60	.00	.02	−.07	.01	.05	.45
10. Did you feel like your vision was sharper or clearer than normal?	−.03	.32	−.15	.18	.23	.29	−.10	.44
68. Did you feel sadness or despair?	−.09	−.05	.73	.11	−.11	.10	.05	.58
70. Did you feel psychological discomfort or significant negative feelings (e.g., intense anxiety or fear, confusion, paranoia)?	.12	−.01	.62	−.20	−.07	.23	.07	.56
40. Did you feel guilt or remorse?	.10	.17	.62	.04	.09	−.06	−.13	.47
32. Did you experience darkness and aloneness?	−.11	.07	.62	.09	−.14	.01	.07	.42
69. Did you cry, or feel an urge to?	.00	−.05	.57	.02	.31	.05	−.06	.46
33. Did you see scenes of violence among humans (e.g., wars, dead bodies, torture, rape, murder)?	.11	.07	.53	.46	−.08	−.13	−.04	.56
51. Did you feel afraid of dying?	.19	−.17	.52	−.26	.00	.13	.32	.58
50. Did you feel like you were dying or that you were dead?	.06	−.13	.42	−.07	.17	.10	.40	.51
28. Did you see visions of the origin of life (e.g., evolution the universe, Earth, and life)?	.08	.05	.02	.73	.05	.10	.03	.70
30. Did you see visions of futuristic technology?	.15	.04	.05	.48	−.06	.06	.08	.34
26. Did you see visions from space (e.g., continents, planets, galaxies, stars)?	.20	.15	−.05	.47	.01	−.03	.16	.45
57. Did you see visions with themes of decay and rebirth?	.18	.06	.28	.45	−.10	.07	.17	.50
29. Did you see visions of indigenous tribes?	.09	.09	−.07	.45	.06	.18	.07	.39
27. Did you see visions of places in different times (e.g., past or future)?	.28	.10	.10	.42	.18	.02	−.02	.57
34. Did these images and visions have meaningful messages associated with them?	.20	.02	.31	.41	.23	−.19	−.05	.53
64. Did these auditory effects have meaningful messages associated with them?	.16	.09	.09	.27	.23	.12	.00	.39
44. Did you feel like an emotional or spiritual weight from the past was lifted?	.05	.09	.02	−.08	.84	.04	−.02	.74
60. Did you feel a greater sense of acceptance of the way things are? (e.g., who you are, your place in things, your past, your present circumstances)	−.05	.10	.00	.07	.79	−.01	−.01	.69
43. Did you feel rejuvenated during or after?	.06	.06	−.08	−.06	.77	−.01	−.05	.59
61. Did you feel an understanding that things in your past have happened for a reason?	−.06	.04	.01	.04	.72	.05	.04	.58
67. Did you feel less anxious?	.08	.10	−.19	−.13	.70	−.03	.04	.50
37. Did you feel that this introspection helped you to process personal issues?	.19	−.07	.06	.06	.68	.12	−.07	.67
41. Did you feel a desire to make amends with people for things that happened in the past?	.07	.15	.22	−.05	.61	−.09	−.06	.47
58. Did you feel a sense of unity or interconnectedness of everything (e.g., the universe, life, humans)?	.04	.02	−.03	.28	.57	.09	.11	.65
52. Did you feel less fear of death?	−.16	.10	.02	.18	.52	−.03	.25	.46
42. Did you feel like you had to surrender to the experience?	.13	.01	.15	−.16	.51	.19	.15	.48
38. Did your memory of past events improve?	.03	.06	.08	.15	.50	.10	−.03	.44
39. Did you relive any emotional significant event? (e.g., trauma, intense joy)	.16	.06	.39	.11	.46	−.02	−.08	.57
45. Did you feel less attached to things (e.g., job, family, death)?	−.07	.00	−.02	.00	.45	.15	.22	.33
36. Did you feel an increased capacity to focus your attention in the present moment?	.11	.05	−.16	.14	.40	.17	−.03	.37
35. Did you feel that your analytic thinking was enhanced?	.10	−.04	.00	.29	.40	.14	−.03	.45
48. Did you feel like some other intelligence was helping to organize or guide your thoughts?	.17	−.11	.02	.30	.36	.12	.18	.50
46. Did you feel distant or detached from your sense of self or your stream of thoughts, like you were able to witness your own thought process?	.10	.01	−.02	.14	.36	.17	.30	.47
59. Did you experience timelessness or irrelevance of time?	.04	.11	.03	.13	.30	.11	.21	.33
6. Did you feel more sensitive to sound?	.02	.02	.04	−.06	−.11	.75	−.05	.53
62. Did you feel that your auditory sensitivity was enhanced?	.18	−.02	−.03	.09	.05	.63	.04	.54
5. Did you feel more sensitive to light or color?	.00	.15	.04	−.06	−.04	.57	−.10	.36
7. Did you feel more sensitive to tastes?	−.10	−.04	.04	.12	.20	.48	−.19	.34
63. Did you hear the sound of buzzing or vibrating?	.09	.06	−.01	−.02	.11	.47	.10	.36
4. Did you like there was feel electricity in your brain or body?	−.02	.06	.07	.09	.10	.46	.11	.36
8. Did your skin feel more sensitive to touch?	−.10	.11	.06	.27	.07	.45	−.03	.39
65. Did you feel like these sounds were coming from somewhere else near you or in the distance, rather than from inside your head?	.37	.00	−.07	.02	−.01	.41	.12	.39
9. Did you feel different senses at the same time (e.g., “feeling the sound”, “seeing the music”)?	−.07	.33	−.08	.26	.01	.40	.08	.50
1. Did you feel like any part of your body was lighter or heavier?	.01	.04	.12	.04	.22	.38	−.02	.31
66. Did you feel like you could see or feel sounds?	−.09	.27	.03	.21	.12	.36	.09	.45
3. Did you notice your hands trembling?	.05	.03	.08	−.16	−.05	.32	.16	.19
2. Did you feel your heart beating faster or slower?	.01	.06	.24	−.16	−.02	.30	.15	.26
54. Did you feel that your body was not yours?	−.25	.14	.10	.17	.22	−.07	.61	.56
49. Did you ever feel completely separated from your body and unaware of its presence?	.14	.00	−.10	.24	.10	−.05	.57	.46
55. Did you feel that the environment was not real?	.12	.14	.03	−.09	−.13	.01	.47	.32
53. Did you feel that things you knew or felt were disappearing?	−.07	.09	.16	.02	.32	.03	.37	.39
47. Did you feel distant or detached from your sense of vision, like you were watching or being watched from somewhere distant?	.09	.04	.02	.25	.20	.10	.33	.39
56. Did you feel that your memory capacities were disturbed?	−.04	.21	.21	−.09	−.19	.09	.32	.29

*Note:* Factor loadings from the rotated matrix (Normalized Direct Oblimin). h²: Communality. NSV: Narrative and Symbolic Visions; VC: Visual Changes; DC: Discomfort and Challenge; C/AV: Cosmic/Archetypal Visions; IPT: Introspection and Personal Transformation; SHPA: Somatosensory Hypersensitivity and Physiological Activation; DI: Dissociation.

Robust goodness‐of‐fit statistics following the LOSEFER correction yielded NNFI = .989, CFI = .991, GFI = .983, and AGFI = .979. The RMSR was.0414—close to the reference value of.0448 according to Kelley [37] —and the WRMR was.0382, confirming an adequate model fit. The seven retained factors demonstrated statistically significant intercorrelations, with coefficients ranging from r = .21 to r = .69 (Table 4).

**Table 4:** Intercorrelations Between the Seven Factors of the IES.

	NSV	VC	DC	C/AV	IPT	SHPA	DI
NSV	1.00	.52	.32	.69	.62	.45	.38
VC		1.00	.21	.46	.45	.50	.39
DC			1.00	.36	.29	.35	.39
C/AV				1.00	.61	.44	.40
IPT					1.00	.51	.44
SHPA						1.00	.42
DI							1.00

*Note:* NSV: Narrative and Symbolic Visions; VC: Visual Changes; DC: Discomfort and Challenge; C/AV: Cosmic/Archetypal Visions; IPT: Introspection and Personal Transformation; SHPA: Somatosensory Hypersensitivity and Physiological Activation; DI: Dissociation. All correlations significant at *p* < .01.

The full 70-item scale showed internal consistency values of α = .948 and ω = .946. However, due to the large number of items, reliability was calculated for each factor separately. Factor 1 (*Narrative and Symbolic Visions*; α_s_ = .87, ω = .87) integrated complex visual experiences with dynamic dream-like sequences, interactions with symbolic entities (masks, characters), and reception of meaningful messages, suggesting structured semiotic processing. Factor 2 (*Visual changes*; α_s_ = .79, ω = .79) described specific visual alterations (geometric patterns, light trails) and heightened perceptual clarity. Factor 3 (*Discomfort and challenge*; α_s_ = .78, ω = .78) grouped intense negative emotions (sadness, guilt, fear of death), experiences of darkness, loneliness, and mortality. Factor 4 (*Cosmic/Archetypal Visions*; α_s_ = .79, ω = .79) encompassed transcendental visionary content, including cosmogonies (universal origins, planetary evolution), tribal symbolism, and decay-rebirth narratives, indicating connections to collective archetypes. Factor 5 (*Introspection and personal transformation*; α_s_ = .91, ω = .91), with excellent reliability, captured identity restructuring mechanisms (acceptance, detachment), introspective clarity, and universal interconnectedness. Factor 6 (*Somatosensory hypersensitivity and physiological activation*; α_s_ = .81, ω = .81) reflected sensory hyperreactivity (auditory, tactile) and autonomic activation (tachycardia, tremors), alongside synesthetic phenomena (e.g., ‘feeling sound’). Factor 7 (*Dissociation*; α_s_ = .65, ω = .65), with moderate internal consistency, included altered self-perception (depersonalization, derealization) and memory distortions. See S3 File.

### Antidependency-agent subscale

The 5-item Antidependency-agent subscale was analyzed separately in the subsample that received ibogaine for the treatment of substance use disorder (n = 118). The optimized parallel analysis suggested unidimensionality, supported by metrics (MIREAL = .144), with its five items explaining 86.5% of the cumulative variance. Goodness-of-fit indices were excellent (GFI = .99; AGFI = .99; CFI = .99). Communalities across items were adequate (.60–.96), construct replicability was high (H-Latent = .98; H-Observed = .743), factor quality and effectiveness were very high (EPTD = 97.9%), and internal consistency values were robust (α_s_ = .96; ω = .96).

### Global experience assessment subscale

An independent analysis was conducted on an 11-item scale where participants evaluated the overall ibogaine experience (see S2 File). The Normed MSA (Measure of Sampling Adequacy) indicated that the polychoric correlation matrix was adequate, with values consistently above.5 for all items (.67–.93). The optimized parallel analysis suggested the scale was unidimensional when using the 95th percentile criterion but bidimensional when applying the mean criterion. The unidimensionality test yielded a value slightly above the expected.3 threshold (MIREAL = .32). Fit indices for the unifactorial solution were adequate (GFI = .95; AGFI = .94; CFI = .99), quality and effectiveness metrics were optimal (EPTD = 95.7%), and internal consistency was acceptable (α_s_ = .90; ω = .92). Based on these results, unidimensionality was retained for the scale.

Additionally, the descriptive analysis of the three questions assessing individual experiences showed:

‘*The most intense effects occurred at*’ (select the correct answer, from the first dose taken): 4.4% reported initial effects within the first hour, 67.3% between the second and fourth hours, 24.6% between the fifth and seventh hours, 3.8% between the eighth and tenth hours.

‘*The most intense effects lasted*’ (select the correct answer, from the first dose taken): 23.0% reported peak intensity within the first three hours, 38.6% between the fourth and sixth hours, 16.0% between the seventh and ninth hours, 1.5% between ten and twelve hours, 11.9% between thirteen and twenty-four hours.

‘*How many hours lasted the entire experience, from the first dose used?*’ (open response): Mean duration: 18.9 hours (SD = 1.8), Range: 0–85 hours.

The Ibogaine Experience Scale (IES) in its final format is presented in the Supporting information (see S2 File).

### Gender differences

The age-adjusted MANCOVA results revealed no statistically significant gender differences in subjective experiences associated with ibogaine consumption overall. However, two exceptions were identified. First, the *Somatosensory hypersensitivity/physiological activation* subscale showed significant differences (*F* = 5.27; *p* = .022; *ω² *= 0.01), with higher scores in women (*M *= 23.77, *SD *= 0.78) compared to men (*M *= 21.77, *SD *= 0.38). Second, the *Antidependency-agent subscale*, analyzed exclusively in the subsample treated for substance use disorders (n = 118), exhibited significant differences (*F* = 4.69; *p* = .011; *ω²* = 0.08), with men scoring higher (*M* = 11.65, *SD* = 4.26) than women (*M* = 8.88, *SD* = 5.89). Adjusted means and complete details are presented in Table 5.

**Table 5:** Adjusted Means, Standard Deviations, and MANCOVA Results for Ibogaine Subjective Experience Scales by Gender.

Scales	Total (n = 498)		Males (n = 404)		Females (n = 94)		MANCOVA		
	M	SD	M	SD	M	SD	F	p	ω^2^
**Narrative and symbolic visions**	19.53	8.46	19.59	0.42	19.14	0.87	0.22	0.64	0.00
**Visual changes**	10.42	4.75	10.51	0.24	10.04	0.49	0.74	0.39	0.00
**Discomfort and challenge**	8.41	5.82	8.48	0.28	8.13	0.59	0.28	0.60	0.00
**Cosmic/Archetypal visions**	7.66	5.88	7.79	0.29	7.05	0.60	1.25	0.26	0.00
**Introspection and personal transformation**	33.14	12.67	33.18	0.62	32.89	1.30	0.04	0.84	0.00
**Somatosensory hypersensitivity/ physiological activation**	22.15	7.71	21.77	0.38	23.77	0.78	5.27	0.02*	0.01
**Dissociation**	5.53	3.85	5.63	0.19	5.04	0.40	1.79	0.18	0.00
	n = 498		n = 404		n = 94				
**Global experience assessment subscale**	28.64	4.15	28.75	3.95	28.19	4.91	0.69	0.501	0.00
	n = 118		n = 101		n = 17				
**Antidependency-agent**	11.25	4.61	11.65	4.26	8.88	5.89	4.69	0.011*	0.08

Note: (*) Mean difference significant at *p* < .05. Marginal means are estimated at the covariate age value (Age = 44.02). *ω*^*2*^ = omega squared for estimating the effect size of the differences.

## Discussion

The present study sought to find the psychometric structure and develop an instrument for capturing the multifaceted phenomenology of ibogaine experiences that we have named the Ibogaine Experience Scale (IES). We departed from a proposal questionnaire with 144 items that, after performing the corresponding psychometric analysis, was reduced to a final set of 70 that accounts for 53.9% of the pooled variance across seven factors, demonstrating excellent fit and reliability indices. To our knowledge, this is the first scale specifically designed to capture the oneiric and multimodal phenomenology of the ibogaine experience, addressing the limitations identified when using instruments developed for serotonergic psychedelics such as the Mystical Experience Questionnaire (MEQ) or the Five Dimensional Altered States of Consciousness (5D-ASC) scale [45,46].

The names of the seven factors were named: 1) Narrative & symbolic visions; 2) Visual changes; 3) Discomfort and challenge; 4) Cosmic/Archetypal visions; 5) Introspection and personal transformation; 6) Somatosensory hypersensitivity and physiological activation; and 7) Dissociation. Plus two supplementary factors named: Antidependency-agent subscale and Global experience assessment subscale. Although further reduction was mathematically possible, eliminating additional items would have risked discarding subtle experiential nuances—such as specific forms of symbolic imagery or bodily sensations—that participants deemed personally meaningful.

Although the IES was developed after a qualitative study that identified several theoretical factors from 20 semi-structured interviews with individuals who had previously undergone a full ibogaine experience [13], the scale also empirically validates the phenomenology that the literature has consistently described regarding ibogaine’s subjective effects. For example, dream-like oneiric visions, buzzing/vibration onset, rapid attenuation of withdrawal/craving, and spiritual or “reset” narratives appear across studies [10–13,47–51]. And Subjective content (visions, autobiographical review, perceived spiritual meaning) is repeatedly linked to longer abstinence or positive mood indices, underscoring the value of structured preparation and post-session integration.

More specifically, Narrative and symbolic visions and Cosmic/Archetypal visions factor describe complex, biographical and trans-personal visions. It matches the “life-review,” “threat-simulation” scenes and archetypal encounters reported in polysubstance-dependent patients [10], in users who self-administered ibogaine in underground settings [47], and in opioid users who had received a single full dose of ibogaine [48]. Visual changes encompasses geometric patterns, color intensification and environmental distortion, the effects that Davis and colleagues (88% “saw visions or visuals”) found to be most prevalent in opioid users treated with ibogaine [12]. Somatosensory hypersensitivity and physiological activation reflects the physical-vegetative component (tachycardia, tremors, buzzing) that stands out both in hospital settings [10,48], and explains why women score higher on this domain. Discomfort and challenge & Dissociation refer to fear, guilt and depersonalization converge with the “challenging states” described by Brown and colleagues [10] and Ozmat and colleagues [49], all of who received a full dose of ibogaine for treating opioid-use disorder and underline the cathartic role of these contents in recovery [12]. Introspection and personal transformation summarizes the processes of insight, self-acceptance and a sense of “reset” that all seven previously cited studies [10–13,47–51] recognize as catalysts of behavioral change (e.g., 67% “gained insightful knowledge” [12]). The almost one-to-one correspondence between IES domains and ethnographic categories supports its content validity and shows that subjective experience—previously captured only narratively—can be quantified reliably (α_total = .95; CFI = .991).

Although most items of the IES loaded strongly (λ > .40) on their primary factor, several displayed substantial cross-loadings that merit theoretical attention rather than methodological concern. Item 48 (“Did these images and visions have meaningful messages associated with them?”) loaded equally on Narrative and Symbolic Visions and on Cosmic/Archetypal Visions, suggesting that symbolic and cosmic motifs may represent intertwined facets of visionary meaning‐making. Item 51 (violent human scenes) loaded significantly on both Discomfort and challenge (λ = .53) and Cosmic/Archetypal visions (λ > .40). This suggests that violent imagery can evoke strong emotional discomfort due to its visceral and disturbing content, while also symbolizing deeper archetypal themes related to the human condition. In this sense, the violent scenes may simultaneously reflect immediate affective challenges (fear, anxiety) and resonate as archetypal symbols of human suffering and conflict, as commonly depicted in mythological or cultural narratives.

Item 91 (“Did you feel like you were dying or that you were dead?”) loaded similarly on Discomfort and challenge and Dissociation, illustrating the dual nature of death-related experiences as both distressing threats and transcendent dissociative states. These overlaps capture real phenomenological complexity rather than scale imperfections.

As just discussed, although some items showed moderate cross-loadings or slightly lower primary factor loadings, they were retained because of their clear theoretical relevance and their concordance with experiential reports documented in qualitative studies. Rather than treating the cross-loadings as methodological flaws, we consider them a reflection of the complex and overlapping nature of the ibogaine experience. As Floyd and Widaman [52] and Tabachnick and Fidell [53] recommend, item retention in exploratory models should be guided by conceptual consistency as much as by statistical criteria. Furthermore, as argued by Clark and Watson [54] and DeVellis [55], discarding items based solely on loading magnitude can result in the loss of important phenomenological content, especially when items uniquely contribute to the interpretive value of a domain. In our analyses, eliminating such items yielded negligible gains in internal consistency, and we prioritized preserving the richness and validity of each subscale.

Reliability analyses confirmed excellent internal consistency for the full 70-item scale (α = .948; ω = .946) and for six of the seven subscales (α/ω between.78 and.91). The moderate reliability of the Dissociation factor (α/ω = .65) likely reflects the heterogeneous nature of depersonalization, derealization, and memory alteration, which may require additional items or refined wording to fully capture. Gender comparisons revealed minimal differences; where significant (e.g., somatosensory hypersensitivity, ω² = .01; antidependency-agent, ω² = .08), effect sizes were small according to Cohen’s benchmarks [56].

Limitations of the scale include a small number of items with low communalities or loadings (e.g., Item 116 on auditory messages), which might benefit from revised phrasing or targeted qualitative exploration. Furthermore, this study’s reliance on convenience sampling from treatment centers may limit generalizability, as participants were self-selected and received care under controlled protocols. The moderate reliability of the Dissociation factor suggests that additional or revised items could better capture its complexity. In addition, the Antidependency-agent sub-scale showed excellent consistency (ω = .96) and sex differences—higher scores in men—suggesting utility for tailoring post-treatment interventions. Importantly, this study introduces the first psychometrically supported instrument specifically designed to assess the acute experiential effects of ibogaine. While the present findings provide initial evidence of construct validity and internal consistency, further research is needed to build on this foundation. For example, additional studies should examine the scale’s convergent and discriminant validity using external measures to clarify how each subscale relates to broader psychological constructs and to strengthen the overall interpretive framework. Finally, although the IES was designed to offer comprehensive coverage of ibogaine’s multifaceted phenomenology, its current length (70 items) may limit its application in time-constrained clinical settings. To address this, future research will focus on developing and validating a short form using Item Response Theory and content-balancing strategies, ensuring usability without compromising theoretical integrity.

The IES has several utilities for both clinical practice and future research. First, the availability of differentiated IES sub-scales enables mechanistic investigations that can identify which phenomenological facets—such as insight-oriented experiences versus archetypal visions—mediate key outcomes, including sustained abstinence and reductions in depressive symptomatology. Second, in the context of clinical trials, the IES can serve as a marker of phenomenological fidelity and be employed as a covariate or moderator when comparing alternative dosing regimens (e.g., flood-dose versus low-dose protocols) or distinct psychological-integration strategies. Finally, because the scale can be administered online within 48 hours of dosing, it lends itself to inclusion in participatory pharmacovigilance platforms, thereby facilitating real-time monitoring and the early detection of uncommon adverse events.

## Conclusion

The IES fills a critical gap in psychedelic research by providing a reliable, comprehensive measure of ibogaine’s unique phenomenology. It offers a psychometrically sound, phenomenologically rich tool for quantifying diverse dimensions of the ibogaine journey. By balancing descriptive depth with rigorous statistical validation, it provides researchers and clinicians with a robust framework for advancing our understanding of the ibogaine experience and the mechanisms underlying its therapeutic effects. Future research should include confirmatory factor analyses in independent and culturally diverse samples. Qualitative follow-up may further refine subscales by incorporating participant narratives that reveal subtle aspects not yet quantified.

## Supporting information

S1 File Items from the preliminary version of the questionnaire. (DOCX)

S2 File Ibogaine Experience Scale (IES). (DOCX)

S3 File Items comprising each factor and factor names. (DOCX)

S4 File Parallel analysis scree plot based on polychoric correlations (PC and FA methods). (DOCX)

S5 File Bootstrap exploratory graph analysis (EGA) and network structure. (DOCX)

S6 File Brief explanation of psychometric statistics used in the analysis. (DOCX)

S7 File Inclusivity in global research questionnaire. (DOCX)

S8 File IES Database. (XLSX)
