# Supplementary material for: The Ibogaine Experience Scale (IES): Development and psychometric properties of a multidimensional measure of ibogaine’s subjective effects
Source: PLoS One. 2025 Oct 13;20(10):e0333296. doi: 10.1371/journal.pone.0333296 (PMC12517489; doi:10.1371/journal.pone.0333296)
Supplement: S1 File — (DOCX) [file pone.0333296.s001.docx]

# S1 Items from the preliminary version of the questionnaire

1. Did you feel like any part of your body was lighter or heavier?

2. Did you feel hot or cold flushes?

3. Did you feel an urge to lie down?

4. Did you feel an urge to close your eyes?

5. Did you feel dizzy or uncoordinated?

6. Did you feel nauseous?

7. Did you vomit?

8. Did you feel your heart beating faster or slower?

9. Did you notice your hands trembling?

10. Did you like there was feel electricity in your brain or body?

11. Did you feel pain in your chest?

12. Did you feel thirsty?

13. Did you feel gastrointestinal effects, such as constipation?

14. Did you feel tremors?

15. Did you feel like you had more physical energy?

16. Did you feel sexually aroused?

17. Did you feel tired or sleepy?

18. Did you feel more sensitive to light or color?

19. Did you feel more sensitive to sound?

20. Did you perceive light differently?

21. Did you perceive sounds differently?

22. Did you feel more sensitive to smells?

23. Did you feel more sensitive to tastes?

24. Did your skin feel more sensitive to touch?

25. Did you feel different senses at the same time (e.g. “feeling the sound”, “seeing the music”)?

26. Did you feel like your vision was sharper or clearer than normal?

27. Did you see patterns or details in the external environment that you could not see before?

28. Did you perceive changes in the colors of the environment (e.g. more vibrant or vivid than usual, or black & white)?

29. Did you see trails of light or other visual distortions (e.g. undulating walls, geometrical patterns, fractals)?

30. Did objects, people, or shadows take on the appearance of other things?

31. Did you see visions of things or characters appear in the room with your eyes open?

32. Did you have visons with closed eyes (e.g. colors, lights, geometrical patterns, fractals)?

33. Did you see dream-like sequences, with visions moving, changing or transforming one into the other?

34. Did any of these sequences involve characters or story-like scenes?

35. Did you see scenes that repeated themselves?

36. Did you have paradoxical images or visions (e.g. light-dark, bad-good, life-death)?

37. Did these dreams have a cartoonish or exaggerated quality?

38. Did you see faces or masks?

39. Did you see or feel the presence of others (e.g., living or deceased people, spirits, archetypes or mythological characters)?

40. Did any of these others interact or communicate with you?

41. Did any of these interactions feel impactful or meaningful?

42. Did you receive any specific information or insights from these interactions?

43. Did you see visions from space (e.g. continents, planets, galaxies, stars)?

44. Did you see visions of places in different times (e.g. past or future)?

45. Did you see visions of the origin of life (e.g. evolution the universe, Earth, and life)?

46. Did you see visions of indigenous tribes?

47. Did you see visions of futuristic technology?

48. Did these images and visions have meaningful messages associated with them?

49. Did you see apocalyptic scenes (e.g. destruction of the planet, catastrophes)?

50. Did you experience darkness and aloneness?

51. Did you see scenes of violence among humans (e.g. wars, dead bodies, torture, rape, murder)?

52. Did these images and visions have meaningful messages associated with them?

53. Did you feel that your analytic thinking was enhanced?

54. Did you feel like your thoughts moved in a way that seemed strange or unfamiliar?

55. Did you feel like your thoughts were racing?

56. Did you feel like things were happening or changing too quickly to remember?

57. Did you feel an increased capacity to focus your attention in the present moment?

58. Did you feel focused on minutia, things that seem extremely small or inconsequential?

59. Did you feel more intensely introspective?

60. Did you feel that this introspection helped you to process personal issues?

61. Did you feel that the experience helped you to solve personal issues?

62. Did you learn things about you that you did not know before?

63. Did your memory of past events improve?

64. Did you relive any emotional significant event? (e.g. trauma, intense joy)

65. Did you have insights?

66. Did you feel that your empathy for people in general increased?

67. Did you feel guilt or remorse?

68. Did you feel more acceptance and forgiveness towards yourself or other people?

69. Did you feel a desire to make amends with people for things that happened in the past?

70. Did you feel more connected to your friends, family members, romantic partners, or people who you work with?

71. Did you feel like you had to surrender to the experience?

72. Did you feel rejuvenated during or after?

73. Did you feel that your mind was clearer?

74. Did you feel spiritually uplifted?

75. Did you feel physically cleansed or relieved from bodily tensions?

76. Did you feel like an emotional or spiritual weight from the past was lifted?

77. Did you feel less attached to things (e. g. job, family, death)?

78. Did you feel like you were a new and better person?

79. Did you feel distant or detached from your sense of self or your stream of thoughts, like you were able to witness your own thought process?

80. Did you feel distant or detached from your sense of vision, like you were watching or being watched from somewhere distant?

81. Did you feel like some other intelligence was helping to organize or guide your thoughts?

82. Did you feel like you were aware of, or present in, two worlds at once?

83. Did you ever feel like you were transported into another world?

84. Did you ever feel completely separated from your body and unaware of its presence?

85. Did you feel emotionally distant from your personal problems, like an observer?

86. Did you feel an increase in your curiosity or openness to new ideas?

87. Did you feel more acceptance towards you and other people?

88. Did you feel in a meditative-like state, with a “quiet mind”?

89. Did you feel an increased capacity to focus your attention in the present moment?

90. Did you feel that you lost your sense of self?

91. Did you feel like you were dying or that you were dead?

92. Did you feel afraid of dying?

93. Did you feel that the experience was like a death-rebirth process?

94. Did you feel less fear of death?

95. Did you feel that things you knew or felt were disappearing?

96. Did you feel that your body was not yours?

97. Did you feel that the environment was not real?

98. Did you feel that your memory capacities were disturbed?

99. Did you see visions with themes of decay and rebirth?

100. Did you feel a sense of unity or interconnectedness of everything (e.g. the universe, life, humans)?

101. Did you feel a deeper understanding of everything (e.g. the organization or structure of the universe, consciousness, everyday life, causalities)?

102. Did you feel more peace and calm?

103. Did you feel more happiness?

104. Did you feel like you were a new and better person?

105. Did you feel a sense of hopefulness?

106. Did you feel like life has a purpose?

107. Did you experience interdependent opposites (light-dark, bad-good, disconnected-connected, life-death)?

108. Did you experience timelessness or irrelevance of time?

109. Did you feel like time was slower or faster?

110. Did you feel a greater sense of acceptance of the way things are? (e.g. who you are, your place in things, your past, your present circumstances)

111. Did you feel an understanding that things in your past have happened for a reason?

112. Did you feel that your auditory sensitivity was enhanced?

113. Did you hear the sound of buzzing or vibrating?

114. Did you feel that your auditory capacities were improved?

115. Did you experience other auditory hallucinations (e.g. hearing a voice, whispering, chanting)?

116. Did these auditory effects have meaningful messages associated with them?

117. Did you feel like these sounds were coming from somewhere else near you or in the distance, rather than from inside your head?

118. Did you feel like you could see or feel sounds?

119. Did you feel reductions in your craving?

120. Did you feel reductions in your withdrawal symptoms?

121. Did you have a better understanding of your reasons to use drugs?

122. Did you feel and increase in your willingness to reduce your drug use?

123. Did you feel more optimistic about your intention of reducing your drug use?

124. Did you feel happier?

125. Did you feel less anxious?

126. Did you feel a sense of awe or wonder?

127. Did you feel sadness or despair?

128. Did you cry, or feel an urge to?

129. Did you feel a sense of detachment from your emotions?

130. Did you feel psychological discomfort or significant negative feelings (e.g. intense anxiety or fear, confusion, paranoia)?

131. In general terms, was the experience challenging?

132. In general terms, were you satisfied with the experience?

133. In general terms, was experience has been useful?

134. In general terms, did the experience meet your expectations?

135. Would you like to repeat the experience?

136. Do you feel as though the benefits obtained will remain for a long period of time?

137. Would you recommend this experience to someone else?

138. In general terms, do you feel that the people taking care of you did a good job?

139. Did you feel safe in the place where you had the experience?

140. Were the facilities in which the experience took place appropriate for this kind of experience?

141. Did you feel as though you retained a sense of control throughout the experience?

142. The most intense effects occurred at (select the correct answer, from the first dose you took):

143. The most intense effects lasted (select the correct answer, from the first dose you took):

144. How many hours lasted the entire experience, from the first dose used?
