# Supplementary material for: The Ibogaine Experience Scale (IES): Development and psychometric properties of a multidimensional measure of ibogaine’s subjective effects
Source: PLoS One. 2025 Oct 13;20(10):e0333296. doi: 10.1371/journal.pone.0333296 (PMC12517489; doi:10.1371/journal.pone.0333296)
Supplement: S2 File — (DOCX) [file pone.0333296.s002.docx]

| 1 | Did you feel like any part of your body was lighter or heavier? |
| --- | --- |
| 2 | Did you feel your heart beating faster or slower? |
| 3 | Did you notice your hands trembling? |
| 4 | Did you like there was feel electricity in your brain or body? |
| 5 | Did you feel more sensitive to light or color? |
| 6 | Did you feel more sensitive to sound? |
| 7 | Did you feel more sensitive to tastes? |
| 8 | Did your skin feel more sensitive to touch? |
| 9 | Did you feel different senses at the same time (eg “feeling the sound”, “seeing the music”)? |
| 10 | Did you feel like your vision was sharper or clearer than normal? |
| 11 | Did you see patterns or details in the external environment that you could not see before? |
| 12 | Did you perceive changes in the colors of the environment (eg more vibrant or vivid than usual, or black & white)? |
| 13 | Did you see trails of light or other visual distortions (eg undulating walls, geometrical patterns, fractals)? |
| 14 | Did objects, people, or shadows take on the appearance of other things? |
| 15 | Did you see visions of things or characters appear in the room with your eyes open? |
| 16 | Did you have visons with closed eyes (eg colors, lights, geometrical patterns, fractals)? |
| 17 | Did you see dream-like sequences, with visions moving, changing or transforming one into the other? |
| 18 | Did any of these sequences involve characters or story-like scenes? |
| 19 | Did you see scenes that repeated themselves? |
| 20 | Did you have paradoxical images or visions (eg light-dark, bad-good, life-death)? |
| 21 | Did these dreams have a cartoonish or exaggerated quality? |
| 22 | Did you see faces or masks? |
| 23 | Did any of these others interact or communicate with you? |
| 24 | Did any of these interactions feel impactful or meaningful? |
| 25 | Did you receive any specific information or insights from these interactions? |
| 26 | Did you see visions from space (eg continents, planets, galaxies, stars)? |
| 27 | Did you see visions of places in different times (eg past or future)? |
| 28 | Did you see visions of the origin of life (eg evolution the universe, Earth, and life)? |
| 29 | Did you see visions of indigenous tribes? |
| 30 | Did you see visions of futuristic technology? |
| 31 | Did these images and visions have meaningful messages associated with them? |
| 32 | Did you experience darkness and aloneness? |
| 33 | Did you see scenes of violence among humans (eg wars, dead bodies, torture, rape, murder)? |
| 34 | Did these images and visions have meaningful messages associated with them? |
| 35 | Did you feel that your analytic thinking was enhanced? |
| 36 | Did you feel an increased capacity to focus your attention in the present moment? |
| 37 | Did you feel that this introspection helped you to process personal issues? |
| 38 | Did your memory of past events improve? |
| 39 | Did you relive any emotional significant event? (eg trauma, intense joy) |
| 40 | Did you feel guilt or remorse? |
| 41 | Did you feel a desire to make amends with people for things that happened in the past? |
| 42 | Did you feel like you had to surrender to the experience? |
| 43 | Did you feel rejuvenated during or after? |
| 44 | Did you feel like an emotional or spiritual weight from the past was lifted? |
| 45 | Did you feel less attached to things (e g job, family, death)? |
| 46 | Did you feel distant or detached from your sense of self or your stream of thoughts, like you were able to witness your own thought process? |
| 47 | Did you feel distant or detached from your sense of vision, like you were watching or being watched from somewhere distant? |
| 48 | Did you feel like some other intelligence was helping to organize or guide your thoughts? |
| 49 | Did you ever feel completely separated from your body and unaware of its presence? |
| 50 | Did you feel like you were dying or that you were dead? |
| 51 | Did you feel afraid of dying? |
| 52 | Did you feel less fear of death? |
| 53 | Did you feel that things you knew or felt were disappearing? |
| 54 | Did you feel that your body was not yours? |
| 55 | Did you feel that the environment was not real? |
| 56 | Did you feel that your memory capacities were disturbed? |
| 57 | Did you see visions with themes of decay and rebirth? |
| 58 | Did you feel a sense of unity or interconnectedness of everything (eg the universe, life, humans)? |
| 59 | Did you experience timelessness or irrelevance of time? |
| 60 | Did you feel a greater sense of acceptance of the way things are? (eg who you are, your place in things, your past, your present circumstances) |
| 61 | Did you feel an understanding that things in your past have happened for a reason? |
| 62 | Did you feel that your auditory sensitivity was enhanced? |
| 63 | Did you hear the sound of buzzing or vibrating? |
| 64 | Did these auditory effects have meaningful messages associated with them? |
| 65 | Did you feel like these sounds were coming from somewhere else near you or in the distance, rather than from inside your head? |
| 66 | Did you feel like you could see or feel sounds? |
| 67 | Did you feel less anxious? |
| 68 | Did you feel sadness or despair? |
| 69 | Did you cry, or feel an urge to? |
| 70 | Did you feel psychological discomfort or significant negative feelings (eg intense anxiety or fear, confusion, paranoia)? |

# S2 Ibogaine Experience Scale (IES)

## Antidependency-subscale

| 71 | Did you feel reductions in your craving? |
| --- | --- |
| 72 | Did you feel reductions in your withdrawal symptoms? |
| 73 | Did you have a better understanding of your reasons to use drugs? |
| 74 | Did you feel and increase in your willingness to reduce your drug use? |
| 75 | Did you feel more optimistic about your intention of reducing your drug use? |

## Global experience assessment subscale

| 76 | In general terms, was the experience challenging? |
| --- | --- |
| 77 | In general terms, were you satisfied with the experience? |
| 78 | In general terms, was experience has been useful? |
| 79 | In general terms, did the experience meet your expectations? |
| 80 | Would you like to repeat the experience? |
| 81 | Do you feel as though the benefits obtained will remain for a long period of time? |
| 82 | Would you recommend this experience to someone else? |
| 83 | In general terms, do you feel that the people taking care of you did a good job? |
| 84 | Did you feel safe in the place where you had the experience? |
| 85 | Were the facilities in which the experience took place appropriate for this kind of experience? |
| 86 | Did you feel as though you retained a sense of control throughout the experience? |
