# Supplementary figures and images for: The Ibogaine Experience Scale (IES): Development and psychometric properties of a multidimensional measure of ibogaine’s subjective effects
Source: PLoS One. 2025 Oct 13;20(10):e0333296. doi: 10.1371/journal.pone.0333296 (PMC12517489; doi:10.1371/journal.pone.0333296)

# S4 Parallel analysis scree plot based on polychoric correlations (PC and FA methods)


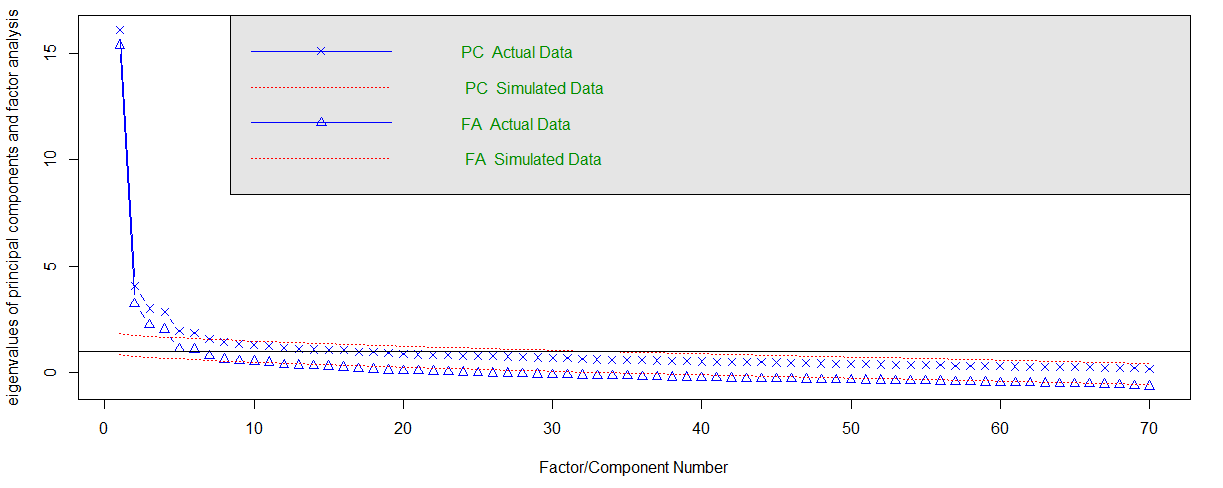

Supplement: S4 File — (DOCX) [file pone.0333296.s004.docx]

# S5 Bootstrap exploratory graph analysis (EGA) and network structure


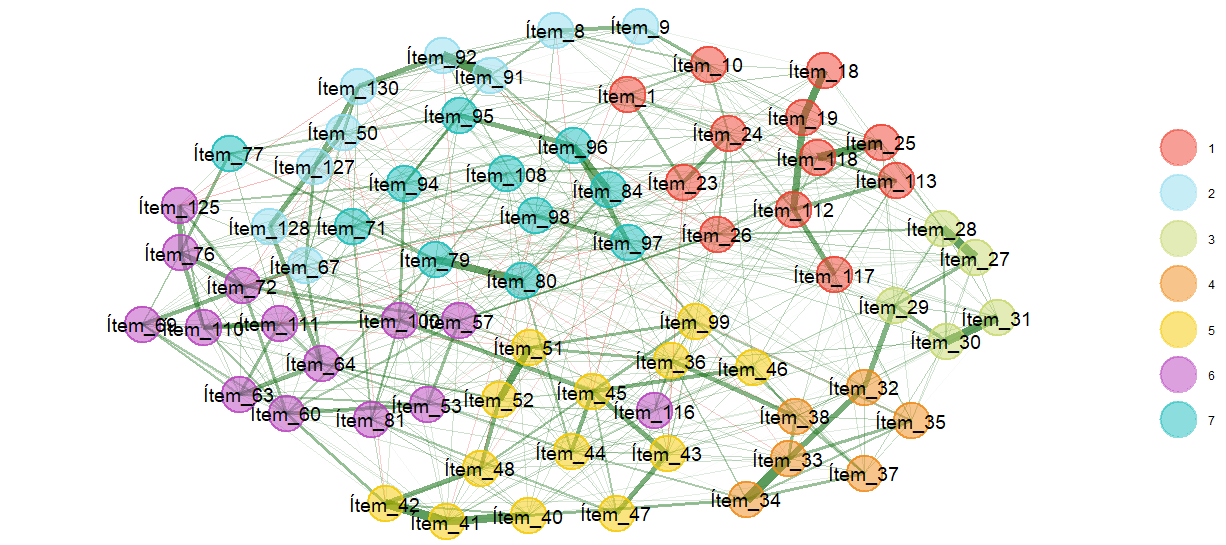

Supplement: S5 File — (DOCX) [file pone.0333296.s005.docx]
