# Supplementary material for: The Ibogaine Experience Scale (IES): Development and psychometric properties of a multidimensional measure of ibogaine’s subjective effects
Source: PLoS One. 2025 Oct 13;20(10):e0333296. doi: 10.1371/journal.pone.0333296 (PMC12517489; doi:10.1371/journal.pone.0333296)
Supplement: S6 File — (DOCX) [file pone.0333296.s006.docx]

| FDI (Factor Determinacy Index) | Indicates how well factor scores reflect the latent variable; values >.90 suggest highly reliable score estimates. |
| --- | --- |
| ORION Marginal Reliability | Measures that marginal reliability of factor scores estimates; values >.80 indicate good precision. |
| SR (Sensitivity Ratio) | Assesses the factor score’s ability to distinguish among individuals. Values >2 are considered acceptable. |
| EPTD (Expected Percentage of True Differences) | Estimates the proportion of true differences among individuals captured by the factor scores; values > 90% indicate excellent reliability. |
| H-index (Construct Replicability) | Reflects how well a latent variable is defined by its indicators and how replicable it would be in other samples. Values > .80 are desirable. |
| MIREAL (Mean of the Item Residual Absolute Loadings) | Evaluates essential unidimensionality; values < .30 suggest a dimension can be treated as essentially unidimensional. |
| MSA (Measure of Sampling Adequacy) | Indicates how appropriate each item is for inclusion in factor analysis; values > .50 are considered acceptable. |
| KMO (Kaiser-Meyer-Olkin Index) | Assesses the overall suitability of the data for factor analysis; values > .80 indicate meritorious or excellent adequacy. |
| RMSR (Root Mean Square Residuals) | An index of model misfit based on residuals; values < .05 suggest a good fit to the data. |
| WRMR (Weighted Root Mean Square Residual) | Similar to RMSR, but adjusts for ordinal data and item weights; values < 1.0 indicate good fit. |
| CFI (Comparative Fit Index) | A goodness-of-fit index comparing the target model to a null model; values > .95 indicate excellent fit, and values > .90 are acceptable. |
| NNFI (Non-Normed Fit Index/Tucker-Lewis Index) | Adjusts for model complexity; values > .95 indicate excellent fit, and values > .90 are acceptable. |
| GFI (Goodness-of-Fit Index) | Measures the proportion of variance accounted for by the estimated population covariance; values > .90 are typically acceptable. |
| AGFI (Adjusted Goodness-of-Fit Index) | A version of GFI adjusted for degrees of freedom; values > .90 are considered acceptable. |

# S6 Brief explanation of psychometric statistics used in the analysis

*Note*. Most of the psychometric indices reported were generated using the software FACTOR v.12.01.02 for Windows (Lorenzo-Seva & Ferrando, 2021). For additional information on these statistics and their interpretation, readers may refer to the program’s documentation and methodological articles by its developers
